# News and Events of the Week

**Published:** 1910-12-31

**Authors:** 


### News and Events of the Week.

Brigade-Surgeon Lieut.-Col. J. R. Murray, M.D., formerly of Rawal Pindi, India, who died on November 17, left estate of the gross value of £30,230 15s. 5d.

Professor Huchard, member of the French Academy of Medicine, editor-in-chief of the *Journal des Praticiens*, and a well-known specialist on cardiac disease, died recently at Paris.

According to a recent report in the *Figaro*, the house at Dôle, where M. Pasteur was born some eighty years ago, will be transformed into a museum. The Municipal Council of Dôle had caused a commemorative tablet to be placed on it some time ago, and now has decided, at a meeting held recently, to purchase this unpretentious property.

Prince Alexander of Teck has received two further promises of £500 each in addition to the five hundred guineas which was promised by a gentleman who desired to remain anonymous, provided that four sums of the same amount were obtained by the end of November. The time has now been extended until the end of the current week to enable Prince Alexander to endeavour to raise the remaining 1,000 guineas.

The second French Congress of Stomatology will take place in Paris from July 31 to August 4, 1911. All foreign practitioners interested in the subject are invited to attend as well as those persons who by reason of their works or their official position are invited to do so by the organising committee. Information can be obtained from the general secretary, Dr. Gires, 4 Rue de Rome, Paris, who will be glad to hear from those desirous of reading papers and of taking part in the discussions.

According to *Le Journal*, the inhabitants of Barca, in the province of Soria, discontented with the doctor provided by the municipality, hinted that he should leave the district. This he refused to do, so an angry crowd besieged the physician's house, taking it by storm, and threatening him with death if he remained a minute longer in the district. There was nothing for the unfortunate disciple of Æsculapius to do but to take the "hint" so forcibly expressed, and he was perforce obliged to set out in search of "fresh fields and pastures new."

We have received the quarterly report on the health of the county borough of Hastings for the three months ending September 30, 1910. A marked decline is noticed in the births registered, there being 128 male and 104 female births, the total 232 giving figures which were twenty-one and thirty-eight less respectively than in 1909 and 1908. The birth-rate was 13.4 per thousand. Deaths totalled 177, against 180 in 1899. The corrected death-rate was 9.9 per thousand. Owing to the small number of deaths from diarrhœal diseases, the zymotic death-rate was only 0.58 per thousand. The most satisfactory feature of the quarter's statistics was the low infantile mortality, only sixty-nine per thousand of births. No epidemic disease was prevalent, and the general health of the borough was good.

Professor Dejerine has been appointed chief neurologist at the Salpêtrière, in succession to the late Professor Raymond.

The ordinary general meeting of the Röntgen Society will be held on Thursday, January 5, 1911, at 20 Hanover Square, at 8.15 p.m., when a paper will be read by Professor Rutherford on "The Radio-activity of Thorium."

Yet another has been added to the already long list of French medical periodicals by the appearance of one entitled *Paris Médical*, a weekly journal produced under the editorship of Professor A. Gilbert. Its aim is to provide for the wants of the clinician. The first number of each month is devoted to some special branch of medicine, and will consist of forty-eight pages of letterpress, as against thirty-two pages for the ordinary numbers. It is published by J. B. Baillière et fils, of Paris, and may be obtained for a subscription of 15 francs per annum. The second number, the only one we have so far seen, is excellent both in subject-matter and in printing, and if the present standard be maintained the success of the venture should be assured.

An inquest held at Derby recently on the body of a collier who had died of injuries received through a fall of binde in the Pentrich Pit, Ripley, elicited the fact that hæmorrhage was the cause of death. A colliery carpenter said that first-aid was rendered at the colliery, and he was instructed to take the injured man to the surgery of Dr. Boyle, the colliery doctor. On the way Neale asked to be taken to see Dr. Hooper, who was the medical officer of the National Deposit Friendly Society, to which Neale belonged. Dr. Hooper saw the man bleeding in his surgery, but declined to attend to him because it was a colliery case. There was twenty minutes' delay in Dr. Hooper's surgery. The Coroner said that, while no medical man was compelled to take any case, he thought it was disgraceful that a doctor should decline to attend to a man who was bleeding in his surgery. The jury added to the verdict a rider embodying this expression of opinion.

We have received the Reports of the Medical Officer of Health and of the Sanitary Inspector of the City of Aberdeen for the month of October 1910, according to which 360 births occurred in the city during the month, equal to an annual rate of 22.8 per 1,000, there being 190 males and 170 females, of whom 330 were legitimate and 30 illegitimate. Marriages totalled 86, giving an annual rate of 5.5 per 1,000; deaths amounted to 178, or an annual rate of 11.3 per 1,000. Of infectious diseases scarlet fever was the most prevalent, 97 cases being reported, of which all recovered. There were 30 cases of diphtheria without a death, two cases of typhoid fever (one of which died), one fatal case of puerperal fever, and 16 cases of erysipelas. Of non-notifiable diseases, four cases of measles, 14 of whooping-cough (of which two were fatal), 15 fatal cases of tuberculosis of the lung and four of other forms of tubercle came under observation. The Sanitary Inspector made 478 inspections with regard to food, and 44 seizures were made of a total weight of 11,527 lb. The health visitors made 325 visits to houses, finding 178 clean, 15 dirty, and 131 medium, one house being revisited and found improved. Infant management accounted for visits to 229 infants, of whom 30 were breast-fed.

